# Supplementary material for: Recent Advancement in Anticancer Compounds from Marine Organisms: Approval, Use and Bioinformatic Approaches to Predict New Targets
Source: Mar Drugs. 2022 Dec 28;21(1):24. doi: 10.3390/md21010024 (PMC9862894; doi:10.3390/md21010024)
Supplement: Supplementary file 1 [file marinedrugs-21-00024-s001.zip › marinedrugs-2019853-supplementary.pdf]

Supplementary Materials

Supplementary Tables

Table S1. Biological activities of Psammaplin A and different marine sources.

Specie	Activity	References
Psammaplin aplysilla revised as *Pseudoceratina purpurea*	Compound discovery	(Quiñoà and Crews 1987)
Psammaplysilla purpurea revised as *Pseudoceratina purpurea*	Derivatives discovery	(Jiménez and Crews 1991)
Association of *Poecillastra sp.* and *Jaspis sp.*	Antibacterial effect against methicillin-resistant *Staphylococcus aureus*	(Kim et al. 1999b)
	Anticancer effect	(Kim et al. 1999a)
Aplysinella rhax	Chitinase Inhibitor	(Tabudravu et al. 2002)
Pseudoceratina purpurea	Histone deacetylase and DNA methyltransferase inhibition	(Piña et al. 2003)
	Inhibitor of aminopeptidase N	(Shim et al. 2004)
	Antiviral	(Salam et al. 2013)
	Anticancer	(Zhou et al. 2018)

Table S2. Most recent clinical approved anticancer compounds from marine species.

Source	Specie	Compound name	Study	Disease	Recruitment status	NCT number
Sponge	*Halicondia okadai*	Eribulin mesylate	Phase III EMBRACE	MCB	Completed	NCT02753595
Sponge	*Pseudoceratina purpurea*	Panobinostat	Phase III PANORAMA-1	RRMM	Completed	NCT01023308
Tunicate	*Ecteinascidia turbinata/ Candidatus Endoecteinascidia frumentensis*	Lurbinectedin	Phase III ATLANTIS	Metastatic Small-Cell Lung Cancer	Completed	NCT02566993
Tunicate	*Ecteinascidia turbinata/ Candidatus Endoecteinascidia frumentensis*	Trabectedin	Phase IV	Advanced Soft Tissue Sarcoma	Completed	NCT01299506
			Phase III	Advanced Liposarcoma and Leiomyosarcoma	Completed	NCT01343277
Tunicate	*Aplidium albicans*	Plitidepsin	Phase III ADMYRE	RRMM	Completed	NCT01102426
Mollusc/Cyanobacteria	*Dolabella auricolaria/ Symploca hynoides, Lyngbya majuscula*	Brentuximab vedotin	Phase II	Hodgkin's Lymphoma, Systemic Anaplastic Large-Cell Lymphoma	Completed	NCT01421667
Mollusc/Cyanobacteria	-	Polatuzumab vedotin	Phase Ib/II	Diffuse large B-cell lymphoma	Completed	NCT01992653
Mollusc/Cyanobacteria	-	Enfortumab vedotin	Phase III	Locally Advanced or Metastatic Urothelial Cancer	Active, not recruiting	NCT03474107
Mollusc/Cyanobacteria	-	Disitamab vedotin	Phase II	Locally Advanced or Metastatic Gastric Cancer	Completed	NCT03556345
			Phase II	Locally Advanced or Metastatic Urothelial Cancer	Unknown	NCT04264936
Mollusc/Cyanobacteria	-	Tisotumab vedotin	Phase II InnovaTV 204	Recurrent Methastatic Cervical Carcinoma	Active, not recruiting	NCT03438396
Mollusc/Cyanobacteria	-	Belantamab mafodotin	Phase II DREAMM-2	RRMM	Active, not recruiting	NCT03525678

Table S3. List of molecules analysed by PASS software, presenting their structural chemical formula, the SMILE format, and the Mol file.

Panobinostat

Chemical structure

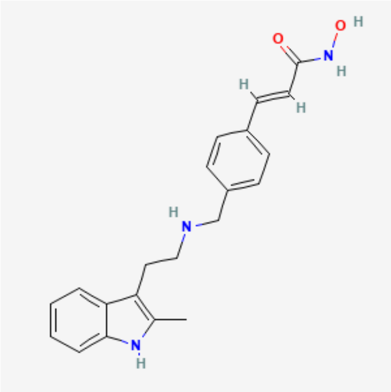

Chemical formula

$C21H23N3O2$

Mol File	Smile format
APtclcactv10112211472D 0 0.00000 0.00000<br><br>49 51 0 0 0 0 0 0 0 0999 V2000<br>6.2619 -3.9838 0.0000 C 0 0 0 0 0 0 0 0<br>0 0 0 0<br>5.2619 -3.9838 0.0000 C 0 0 0 0 0 0 0 0<br>0 0 0 0<br>4.6783 -3.1791 0.0000 C 0 0 0 0 0 0 0 0<br>0 0 0 0<br>3.7321 -3.4838 0.0000 C 0 0 0 0 0 0 0 0<br>0 0 0 0<br>2.8660 -2.9838 0.0000 C 0 0 0 0 0 0 0 0<br>0 0 0 0<br>2.0000 -3.4838 0.0000 C 0 0 0 0 0 0 0 0<br>0 0 0 0<br>2.0000 -4.4838 0.0000 C 0 0 0 0 0 0 0 0<br>0 0 0 0<br>2.8660 -4.9838 0.0000 C 0 0 0 0 0 0 0 0<br>0 0 0 0	CC1=C(C2=CC=CC=C2N1)CCNCC3=CC=C<br>(C=C3)C=CC(=O)NO

3.7321 -4.4838 0.0000 C 0 0 0 0 0 0 0 0
0 0 0 0
4.6783 -4.7885 0.0000 N 0 0 0 0 0 0 0 0
0 0 0 0
4.9889 -2.2285 0.0000 C 0 0 0 0 0 0 0 0
0 0 0 0
5.9674 -2.0223 0.0000 C 0 0 0 0 0 0 0 0
0 0 0 0
6.2781 -1.0718 0.0000 N 0 0 0 0 0 0 0 0
0 0 0 0
7.2566 -0.8656 0.0000 C 0 0 0 0 0 0 0 0
0 0 0 0
7.5673 0.0849 0.0000 C 0 0 0 0 0 0 0 0
0 0 0 0
8.5458 0.2912 0.0000 C 0 0 0 0 0 0 0 0
0 0 0 0
8.8564 1.2417 0.0000 C 0 0 0 0 0 0 0 0
0 0 0 0
8.1886 1.9860 0.0000 C 0 0 0 0 0 0 0 0
0 0 0 0
7.2101 1.7798 0.0000 C 0 0 0 0 0 0 0 0
0 0 0 0
6.8994 0.8292 0.0000 C 0 0 0 0 0 0 0 0
0 0 0 0
8.4993 2.9365 0.0000 C 0 0 0 0 0 0 0 0
0 0 0 0
9.4778 3.1427 0.0000 C 0 0 0 0 0 0 0 0
0 0 0 0
9.7884 4.0932 0.0000 C 0 0 0 0 0 0 0 0
0 0 0 0
9.1206 4.8375 0.0000 O 0 0 0 0 0 0 0 0
0 0 0 0
10.7669 4.2995 0.0000 N 0 0 0 0 0 0 0 0
0 0 0 0
11.0776 5.2500 0.0000 O 0 0 0 0 0 0 0 0
0 0 0 0
6.2619 -4.6038 0.0000 H 0 0 0 0 0 0 0 0
0 0 0 0
6.8819 -3.9838 0.0000 H 0 0 0 0 0 0 0 0
0 0 0 0
6.2619 -3.3638 0.0000 H 0 0 0 0 0 0 0 0
0 0 0 0
2.8660 -2.3638 0.0000 H 0 0 0 0 0 0 0 0
0 0 0 0
1.4631 -3.1738 0.0000 H 0 0 0 0 0 0 0 0
0 0 0 0
1.4631 -4.7938 0.0000 H 0 0 0 0 0 0 0 0
0 0 0 0
2.8660 -5.6038 0.0000 H 0 0 0 0 0 0 0 0
0 0 0 0
4.8709 -5.3778 0.0000 H 0 0 0 0 0 0 0 0
0 0 0 0

```
    4.9684   -1.6089    0.0000 H   0  0  0  0  0  0  0  0
0  0  0  0
    4.3751   -2.1412    0.0000 H   0  0  0  0  0  0  0  0
0  0  0  0
    5.9880   -2.6420    0.0000 H   0  0  0  0  0  0  0  0
0  0  0  0
    6.5813   -2.1097    0.0000 H   0  0  0  0  0  0  0  0
0  0  0  0
    5.8640   -0.6103    0.0000 H   0  0  0  0  0  0  0  0
0  0  0  0
    7.2772   -1.4852    0.0000 H   0  0  0  0  0  0  0  0
0  0  0  0
    7.8704   -0.9529    0.0000 H   0  0  0  0  0  0  0  0
0  0  0  0
    8.9598   -0.1703    0.0000 H   0  0  0  0  0  0  0  0
0  0  0  0
    9.4631    1.3695    0.0000 H   0  0  0  0  0  0  0  0
0  0  0  0
    6.7960    2.2412    0.0000 H   0  0  0  0  0  0  0  0
0  0  0  0
    6.2928    0.7014    0.0000 H   0  0  0  0  0  0  0  0
0  0  0  0
    8.0852    3.3980    0.0000 H   0  0  0  0  0  0  0  0
0  0  0  0
    9.8918    2.6813    0.0000 H   0  0  0  0  0  0  0  0
0  0  0  0
   11.1810    3.8380    0.0000 H   0  0  0  0  0  0  0  0
0  0  0  0
   11.6843    5.3778    0.0000 H   0  0  0  0  0  0  0  0
0  0  0  0
  1  2  1  0  0  0  0
  2  3  2  0  0  0  0
  3  4  1  0  0  0  0
  4  5  2  0  0  0  0
  5  6  1  0  0  0  0
  6  7  2  0  0  0  0
  7  8  1  0  0  0  0
  8  9  2  0  0  0  0
  4  9  1  0  0  0  0
  9 10  1  0  0  0  0
  2 10  1  0  0  0  0
  3 11  1  0  0  0  0
 11 12  1  0  0  0  0
 12 13  1  0  0  0  0
 13 14  1  0  0  0  0
 14 15  1  0  0  0  0
 15 16  2  0  0  0  0
 16 17  1  0  0  0  0
 17 18  2  0  0  0  0
 18 19  1  0  0  0  0
 19 20  2  0  0  0  0
 15 20  1  0  0  0  0
```

18 21 1 0 0 0 0
21 22 2 3 0 0 0
22 23 1 0 0 0 0
23 24 2 0 0 0 0
23 25 1 0 0 0 0
25 26 1 0 0 0 0
 1 27 1 0 0 0 0
 1 28 1 0 0 0 0
 1 29 1 0 0 0 0
 5 30 1 0 0 0 0
 6 31 1 0 0 0 0
 7 32 1 0 0 0 0
 8 33 1 0 0 0 0
10 34 1 0 0 0 0
11 35 1 0 0 0 0
11 36 1 0 0 0 0
12 37 1 0 0 0 0
12 38 1 0 0 0 0
13 39 1 0 0 0 0
14 40 1 0 0 0 0
14 41 1 0 0 0 0
16 42 1 0 0 0 0
17 43 1 0 0 0 0
19 44 1 0 0 0 0
20 45 1 0 0 0 0
21 46 1 0 0 0 0
22 47 1 0 0 0 0
25 48 1 0 0 0 0
26 49 1 0 0 0 0
M END
$$$$

Plitidepsin

Chemical structure

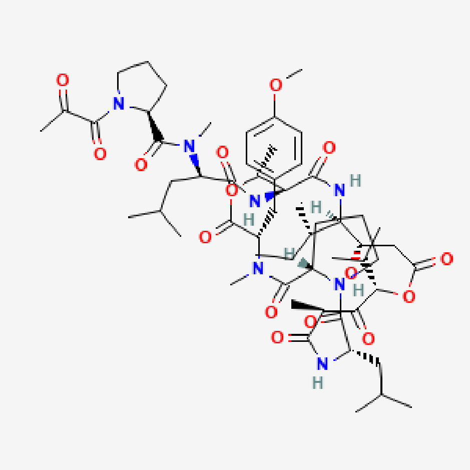

Chemical formula

$C_{57}H_{87}N_{7}O_{15}$

Mol file

```
APtclcactv10052204412D 0   0.00000     0.00000


166169  0  0  0  0  0  0  0  0  0999 V2000
    9.1558    1.1602    0.0000 C   0  0  0  0  0  0  0  0  0  0  0  0
    8.1567    1.1166    0.0000 C   0  0  0  0  0  0  0  0  0  0  0  0
    7.6950    0.2296    0.0000 C   0  0  0  0  0  0  0  0  0  0  0  0
    6.7711   -0.1530    0.0000 C   0  0  0  0  0  0  0  0  0  0  0  0
    8.2323   -0.6138    0.0000 C   0  0  0  0  0  0  0  0  0  0  0  0
    8.9394   -1.3209    0.0000 C   0  0  0  0  0  0  0  0  0  0  0  0
    9.9394   -1.3209    0.0000 C   0  0  0  0  0  0  0  0  0  0  0  0
   10.6465   -2.0280    0.0000 C   0  0  0  0  0  0  0  0  0  0  0  0
   11.6124   -1.7692    0.0000 O   0  0  0  0  0  0  0  0  0  0  0  0
   10.3877   -2.9940    0.0000 O   0  0  0  0  0  0  0  0  0  0  0  0
    9.4218   -2.7351    0.0000 C   0  0  0  0  0  0  0  0  0  0  0  0
    8.7147   -3.4423    0.0000 C   0  0  0  0  0  0  0  0  0  0  0  0
    9.0973   -4.3661    0.0000 O   0  0  0  0  0  0  0  0  0  0  0  0
    7.7147   -3.4423    0.0000 C   0  0  0  0  0  0  0  0  0  0  0  0
    7.2147   -4.3083    0.0000 C   0  0  0  0  0  0  0  0  0  0  0  0
    6.2147   -4.3083    0.0000 O   0  0  0  0  0  0  0  0  0  0  0  0
    7.7147   -5.1743    0.0000 N   0  0  0  0  0  0  0  0  0  0  0  0
    8.5807   -4.6743    0.0000 C   0  0  0  0  0  0  0  0  0  0  0  0
    8.2323   -3.6138    0.0000 C   0  0  0  0  0  0  0  0  0  0  0  0
    7.2479   -3.7900    0.0000 O   0  0  0  0  0  0  0  0  0  0  0  0
    8.2323   -2.6138    0.0000 N   0  0  0  0  0  0  0  0  0  0  0  0
    9.4805   -1.7860    0.0000 C   0  0  0  0  0  0  0  0  0  0  0  0
    8.9392   -0.3894    0.0000 C   0  0  0  0  0  0  0  0  0  0  0  0
    7.4591   -0.6189    0.0000 C   0  0  0  0  0  0  0  0  0  0  0  0
    7.3663   -2.1138    0.0000 C   0  0  0  0  0  0  0  0  0  0  0  0
    6.5002   -2.6138    0.0000 C   0  0  0  0  0  0  0  0  0  0  0  0
```

SMILE format

CCC(C)C1C(CC(=O)OC(C(=O)C(C(=O)NC(C(=O)N2C
CCC2C(=O)N(C(C(=O)OC(C(C(=O)N1)NC(=O)C(CC(
C)C)N(C)C(=O)C3CCCN3C(=O)C(=O)C)C)CC4=CC=
C(C=C4)OC)C)CC(C)C)C)C(C)C)O

6.5002	-3.6138	0.0000	O	0	0	0	0	0	0	0	0	0	0	0	0
5.6342	-2.1138	0.0000	N	0	0	0	0	0	0	0	0	0	0	0	0
5.6342	-1.1138	0.0000	C	0	0	0	0	0	0	0	0	0	0	0	0
4.7682	-0.6138	0.0000	C	0	0	0	0	0	0	0	0	0	0	0	0
3.9022	-1.1138	0.0000	O	0	0	0	0	0	0	0	0	0	0	0	0
4.7682	0.3862	0.0000	O	0	0	0	0	0	0	0	0	0	0	0	0
5.6342	0.8862	0.0000	C	0	0	0	0	0	0	0	0	0	0	0	0
6.5002	0.3862	0.0000	C	0	0	0	0	0	0	0	0	0	0	0	0
7.3663	0.8862	0.0000	C	0	0	0	0	0	0	0	0	0	0	0	0
7.3663	1.8862	0.0000	O	0	0	0	0	0	0	0	0	0	0	0	0
8.2323	0.3862	0.0000	N	0	0	0	0	0	0	0	0	0	0	0	0
5.5002	0.3862	0.0000	N	0	0	0	0	0	0	0	0	0	0	0	0
5.0003	1.2522	0.0000	C	0	0	0	0	0	0	0	0	0	0	0	0
5.1741	2.2370	0.0000	O	0	0	0	0	0	0	0	0	0	0	0	0
4.0003	1.2524	0.0000	C	0	0	0	0	0	0	0	0	0	0	0	0
3.5001	0.3865	0.0000	C	0	0	0	0	0	0	0	0	0	0	0	0
2.5001	0.3867	0.0000	C	0	0	0	0	0	0	0	0	0	0	0	0
2.0000	-0.4793	0.0000	C	0	0	0	0	0	0	0	0	0	0	0	0
2.0002	1.2527	0.0000	C	0	0	0	0	0	0	0	0	0	0	0	0
3.5004	2.1185	0.0000	N	0	0	0	0	0	0	0	0	0	0	0	0
2.5004	2.1186	0.0000	C	0	0	0	0	0	0	0	0	0	0	0	0
4.0005	2.9844	0.0000	C	0	0	0	0	0	0	0	0	0	0	0	0
5.0005	2.9842	0.0000	O	0	0	0	0	0	0	0	0	0	0	0	0
3.5006	3.8505	0.0000	C	0	0	0	0	0	0	0	0	0	0	0	0
2.5061	3.9552	0.0000	C	0	0	0	0	0	0	0	0	0	0	0	0
2.2984	4.9334	0.0000	C	0	0	0	0	0	0	0	0	0	0	0	0
3.1644	5.4332	0.0000	C	0	0	0	0	0	0	0	0	0	0	0	0
3.9075	4.7640	0.0000	N	0	0	0	0	0	0	0	0	0	0	0	0
4.8857	4.9717	0.0000	C	0	0	0	0	0	0	0	0	0	0	0	0
5.5546	4.2285	0.0000	O	0	0	0	0	0	0	0	0	0	0	0	0
5.1948	5.9227	0.0000	C	0	0	0	0	0	0	0	0	0	0	0	0
6.1730	6.1305	0.0000	O	0	0	0	0	0	0	0	0	0	0	0	0
4.5258	6.6660	0.0000	C	0	0	0	0	0	0	0	0	0	0	0	0
5.9762	1.8259	0.0000	C	0	0	0	0	0	0	0	0	0	0	0	0
5.9762	-2.0535	0.0000	C	0	0	0	0	0	0	0	0	0	0	0	0
5.3335	-2.8196	0.0000	C	0	0	0	0	0	0	0	0	0	0	0	0
4.3487	-2.6460	0.0000	C	0	0	0	0	0	0	0	0	0	0	0	0
3.7059	-3.4120	0.0000	C	0	0	0	0	0	0	0	0	0	0	0	0
4.0479	-4.3517	0.0000	C	0	0	0	0	0	0	0	0	0	0	0	0
5.0327	-4.5253	0.0000	C	0	0	0	0	0	0	0	0	0	0	0	0
5.6755	-3.7593	0.0000	C	0	0	0	0	0	0	0	0	0	0	0	0
3.4051	-5.1177	0.0000	O	0	0	0	0	0	0	0	0	0	0	0	0
2.4203	-4.9441	0.0000	C	0	0	0	0	0	0	0	0	0	0	0	0
4.6342	-2.1138	0.0000	C	0	0	0	0	0	0	0	0	0	0	0	0
9.4887	-5.0933	0.0000	C	0	0	0	0	0	0	0	0	0	0	0	0
9.5797	-6.0892	0.0000	C	0	0	0	0	0	0	0	0	0	0	0	0
10.4877	-6.5082	0.0000	C	0	0	0	0	0	0	0	0	0	0	0	0
8.7628	-6.6660	0.0000	C	0	0	0	0	0	0	0	0	0	0	0	0
7.0719	-2.6762	0.0000	C	0	0	0	0	0	0	0	0	0	0	0	0
8.9991	-1.8288	0.0000	C	0	0	0	0	0	0	0	0	0	0	0	0
9.4852	-0.7866	0.0000	C	0	0	0	0	0	0	0	0	0	0	0	0
8.0332	-1.5700	0.0000	C	0	0	0	0	0	0	0	0	0	0	0	0

8.5567	-2.2448	0.0000	O	0	0	0	0	0	0	0	0	0	0	0	0
9.1828	0.5408	0.0000	H	0	0	0	0	0	0	0	0	0	0	0	0
9.7752	1.1872	0.0000	H	0	0	0	0	0	0	0	0	0	0	0	0
9.1288	1.7796	0.0000	H	0	0	0	0	0	0	0	0	0	0	0	0
8.2377	1.7313	0.0000	H	0	0	0	0	0	0	0	0	0	0	0	0
7.5654	1.3030	0.0000	H	0	0	0	0	0	0	0	0	0	0	0	0
7.2762	0.6867	0.0000	H	0	0	0	0	0	0	0	0	0	0	0	0
6.5339	0.4198	0.0000	H	0	0	0	0	0	0	0	0	0	0	0	0
6.1983	-0.3902	0.0000	H	0	0	0	0	0	0	0	0	0	0	0	0
7.0083	-0.7258	0.0000	H	0	0	0	0	0	0	0	0	0	0	0	0
8.0588	-0.0186	0.0000	H	0	0	0	0	0	0	0	0	0	0	0	0
9.2839	-1.8364	0.0000	H	0	0	0	0	0	0	0	0	0	0	0	0
10.4763	-1.0109	0.0000	H	0	0	0	0	0	0	0	0	0	0	0	0
9.7789	-0.7221	0.0000	H	0	0	0	0	0	0	0	0	0	0	0	0
9.8203	-2.2602	0.0000	H	0	0	0	0	0	0	0	0	0	0	0	0
7.9767	-2.8803	0.0000	H	0	0	0	0	0	0	0	0	0	0	0	0
7.5542	-5.7732	0.0000	H	0	0	0	0	0	0	0	0	0	0	0	0
8.6089	-5.2937	0.0000	H	0	0	0	0	0	0	0	0	0	0	0	0
9.7852	-2.3259	0.0000	H	0	0	0	0	0	0	0	0	0	0	0	0
10.0410	-1.5209	0.0000	H	0	0	0	0	0	0	0	0	0	0	0	0
9.5319	-0.2075	0.0000	H	0	0	0	0	0	0	0	0	0	0	0	0
8.8004	0.2149	0.0000	H	0	0	0	0	0	0	0	0	0	0	0	0
7.4084	-0.0010	0.0000	H	0	0	0	0	0	0	0	0	0	0	0	0
6.8392	-0.6251	0.0000	H	0	0	0	0	0	0	0	0	0	0	0	0
6.8392	-1.7873	0.0000	H	0	0	0	0	0	0	0	0	0	0	0	0
6.1092	-0.7153	0.0000	H	0	0	0	0	0	0	0	0	0	0	0	0
5.1593	1.2847	0.0000	H	0	0	0	0	0	0	0	0	0	0	0	0
6.6607	-0.2127	0.0000	H	0	0	0	0	0	0	0	0	0	0	0	0
8.7692	0.6962	0.0000	H	0	0	0	0	0	0	0	0	0	0	0	0
5.1903	-0.1508	0.0000	H	0	0	0	0	0	0	0	0	0	0	0	0
4.3104	1.7893	0.0000	H	0	0	0	0	0	0	0	0	0	0	0	0
3.3923	-0.2241	0.0000	H	0	0	0	0	0	0	0	0	0	0	0	0
4.0827	0.1743	0.0000	H	0	0	0	0	0	0	0	0	0	0	0	0
2.8102	0.9236	0.0000	H	0	0	0	0	0	0	0	0	0	0	0	0
1.4631	-0.1692	0.0000	H	0	0	0	0	0	0	0	0	0	0	0	0
1.6899	-1.0162	0.0000	H	0	0	0	0	0	0	0	0	0	0	0	0
2.5369	-0.7894	0.0000	H	0	0	0	0	0	0	0	0	0	0	0	0
2.5372	1.5626	0.0000	H	0	0	0	0	0	0	0	0	0	0	0	0
1.6903	1.7896	0.0000	H	0	0	0	0	0	0	0	0	0	0	0	0
1.4633	0.9427	0.0000	H	0	0	0	0	0	0	0	0	0	0	0	0
2.5004	2.7386	0.0000	H	0	0	0	0	0	0	0	0	0	0	0	0
1.8804	2.1187	0.0000	H	0	0	0	0	0	0	0	0	0	0	0	0
2.5003	1.4986	0.0000	H	0	0	0	0	0	0	0	0	0	0	0	0
4.1197	3.8828	0.0000	H	0	0	0	0	0	0	0	0	0	0	0	0
1.8895	3.8905	0.0000	H	0	0	0	0	0	0	0	0	0	0	0	0
2.5060	3.3352	0.0000	H	0	0	0	0	0	0	0	0	0	0	0	0
2.0463	5.4998	0.0000	H	0	0	0	0	0	0	0	0	0	0	0	0
1.7087	4.7419	0.0000	H	0	0	0	0	0	0	0	0	0	0	0	0
3.6252	5.8480	0.0000	H	0	0	0	0	0	0	0	0	0	0	0	0
2.8001	5.9348	0.0000	H	0	0	0	0	0	0	0	0	0	0	0	0
4.9867	7.0808	0.0000	H	0	0	0	0	0	0	0	0	0	0	0	0
4.1111	7.1268	0.0000	H	0	0	0	0	0	0	0	0	0	0	0	0

```
   4.0650    6.2512    0.0000 H   0  0  0  0  0  0  0  0  0  0  0  0
   6.5589    1.6138    0.0000 H   0  0  0  0  0  0  0  0  0  0  0  0
   6.1883    2.4085    0.0000 H   0  0  0  0  0  0  0  0  0  0  0  0
   5.3936    2.0379    0.0000 H   0  0  0  0  0  0  0  0  0  0  0  0
   6.3748   -2.5285    0.0000 H   0  0  0  0  0  0  0  0  0  0  0  0
   6.5132   -1.7435    0.0000 H   0  0  0  0  0  0  0  0  0  0  0  0
   4.1366   -2.0634    0.0000 H   0  0  0  0  0  0  0  0  0  0  0  0
   3.0953   -3.3043    0.0000 H   0  0  0  0  0  0  0  0  0  0  0  0
   5.2447   -5.1079    0.0000 H   0  0  0  0  0  0  0  0  0  0  0  0
   6.2861   -3.8669    0.0000 H   0  0  0  0  0  0  0  0  0  0  0  0
   2.5280   -4.3335    0.0000 H   0  0  0  0  0  0  0  0  0  0  0  0
   1.8098   -4.8365    0.0000 H   0  0  0  0  0  0  0  0  0  0  0  0
   2.3127   -5.5547    0.0000 H   0  0  0  0  0  0  0  0  0  0  0  0
   4.6342   -1.4938    0.0000 H   0  0  0  0  0  0  0  0  0  0  0  0
   4.0142   -2.1138    0.0000 H   0  0  0  0  0  0  0  0  0  0  0  0
   4.6342   -2.7338    0.0000 H   0  0  0  0  0  0  0  0  0  0  0  0
  10.1065   -5.1450    0.0000 H   0  0  0  0  0  0  0  0  0  0  0  0
   9.6468   -4.4938    0.0000 H   0  0  0  0  0  0  0  0  0  0  0  0
   9.0168   -5.8294    0.0000 H   0  0  0  0  0  0  0  0  0  0  0  0
  10.2279   -7.0712    0.0000 H   0  0  0  0  0  0  0  0  0  0  0  0
  11.0506   -6.7680    0.0000 H   0  0  0  0  0  0  0  0  0  0  0  0
  10.7475   -5.9453    0.0000 H   0  0  0  0  0  0  0  0  0  0  0  0
   8.4052   -6.1595    0.0000 H   0  0  0  0  0  0  0  0  0  0  0  0
   8.2564   -7.0236    0.0000 H   0  0  0  0  0  0  0  0  0  0  0  0
   9.1205   -7.1725    0.0000 H   0  0  0  0  0  0  0  0  0  0  0  0
   7.5468   -2.2777    0.0000 H   0  0  0  0  0  0  0  0  0  0  0  0
   6.6733   -2.2013    0.0000 H   0  0  0  0  0  0  0  0  0  0  0  0
   6.5969   -3.0747    0.0000 H   0  0  0  0  0  0  0  0  0  0  0  0
   9.6191   -1.8288    0.0000 H   0  0  0  0  0  0  0  0  0  0  0  0
  10.0471   -1.0486    0.0000 H   0  0  0  0  0  0  0  0  0  0  0  0
   9.7472   -0.2247    0.0000 H   0  0  0  0  0  0  0  0  0  0  0  0
   8.9232   -0.5246    0.0000 H   0  0  0  0  0  0  0  0  0  0  0  0
   8.1937   -0.9711    0.0000 H   0  0  0  0  0  0  0  0  0  0  0  0
   7.4343   -1.4096    0.0000 H   0  0  0  0  0  0  0  0  0  0  0  0
   7.8728   -2.1689    0.0000 H   0  0  0  0  0  0  0  0  0  0  0  0
   8.8430   -2.7948    0.0000 H   0  0  0  0  0  0  0  0  0  0  0  0
  1  2  1  0  0  0  0
  2  3  1  0  0  0  0
  3  4  1  0  0  0  0
  3  5  1  0  0  0  0
  5  6  1  0  0  0  0
  6  7  1  0  0  0  0
  7  8  1  0  0  0  0
  8  9  2  0  0  0  0
  8 10  1  0  0  0  0
 10 11  1  0  0  0  0
 11 12  1  0  0  0  0
 12 13  2  0  0  0  0
 12 14  1  0  0  0  0
 14 15  1  0  0  0  0
 15 16  2  0  0  0  0
 15 17  1  0  0  0  0
```

17	18	1	0	0	0	0
18	19	1	0	0	0	0
19	20	2	0	0	0	0
19	21	1	0	0	0	0
21	22	1	0	0	0	0
22	23	1	0	0	0	0
23	24	1	0	0	0	0
24	25	1	0	0	0	0
21	25	1	0	0	0	0
25	26	1	0	0	0	0
26	27	2	0	0	0	0
26	28	1	0	0	0	0
28	29	1	0	0	0	0
29	30	1	0	0	0	0
30	31	2	0	0	0	0
30	32	1	0	0	0	0
32	33	1	0	0	0	0
33	34	1	0	0	0	0
34	35	1	0	0	0	0
35	36	2	0	0	0	0
35	37	1	0	0	0	0
5	37	1	0	0	0	0
34	38	1	0	0	0	0
38	39	1	0	0	0	0
39	40	2	0	0	0	0
39	41	1	0	0	0	0
41	42	1	0	0	0	0
42	43	1	0	0	0	0
43	44	1	0	0	0	0
43	45	1	0	0	0	0
41	46	1	0	0	0	0
46	47	1	0	0	0	0
46	48	1	0	0	0	0
48	49	2	0	0	0	0
48	50	1	0	0	0	0
50	51	1	0	0	0	0
51	52	1	0	0	0	0
52	53	1	0	0	0	0
53	54	1	0	0	0	0
50	54	1	0	0	0	0
54	55	1	0	0	0	0
55	56	2	0	0	0	0
55	57	1	0	0	0	0
57	58	2	0	0	0	0
57	59	1	0	0	0	0
33	60	1	0	0	0	0
29	61	1	0	0	0	0
61	62	1	0	0	0	0
62	63	2	0	0	0	0
63	64	1	0	0	0	0
64	65	2	0	0	0	0
65	66	1	0	0	0	0

66 67 2 0 0 0 0
62 67 1 0 0 0 0
65 68 1 0 0 0 0
68 69 1 0 0 0 0
28 70 1 0 0 0 0
18 71 1 0 0 0 0
71 72 1 0 0 0 0
72 73 1 0 0 0 0
72 74 1 0 0 0 0
14 75 1 0 0 0 0
11 76 1 0 0 0 0
76 77 1 0 0 0 0
76 78 1 0 0 0 0
6 79 1 0 0 0 0
1 80 1 0 0 0 0
1 81 1 0 0 0 0
1 82 1 0 0 0 0
2 83 1 0 0 0 0
2 84 1 0 0 0 0
3 85 1 0 0 0 0
4 86 1 0 0 0 0
4 87 1 0 0 0 0
4 88 1 0 0 0 0
5 89 1 0 0 0 0
6 90 1 0 0 0 0
7 91 1 0 0 0 0
7 92 1 0 0 0 0
11 93 1 0 0 0 0
14 94 1 0 0 0 0
17 95 1 0 0 0 0
18 96 1 0 0 0 0
22 97 1 0 0 0 0
22 98 1 0 0 0 0
23 99 1 0 0 0 0
23100 1 0 0 0 0
24101 1 0 0 0 0
24102 1 0 0 0 0
25103 1 0 0 0 0
29104 1 0 0 0 0
33105 1 0 0 0 0
34106 1 0 0 0 0
37107 1 0 0 0 0
38108 1 0 0 0 0
41109 1 0 0 0 0
42110 1 0 0 0 0
42111 1 0 0 0 0
43112 1 0 0 0 0
44113 1 0 0 0 0
44114 1 0 0 0 0
44115 1 0 0 0 0
45116 1 0 0 0 0
45117 1 0 0 0 0

45118	1	0	0	0	0
47119	1	0	0	0	0
47120	1	0	0	0	0
47121	1	0	0	0	0
50122	1	0	0	0	0
51123	1	0	0	0	0
51124	1	0	0	0	0
52125	1	0	0	0	0
52126	1	0	0	0	0
53127	1	0	0	0	0
53128	1	0	0	0	0
59129	1	0	0	0	0
59130	1	0	0	0	0
59131	1	0	0	0	0
60132	1	0	0	0	0
60133	1	0	0	0	0
60134	1	0	0	0	0
61135	1	0	0	0	0
61136	1	0	0	0	0
63137	1	0	0	0	0
64138	1	0	0	0	0
66139	1	0	0	0	0
67140	1	0	0	0	0
69141	1	0	0	0	0
69142	1	0	0	0	0
69143	1	0	0	0	0
70144	1	0	0	0	0
70145	1	0	0	0	0
70146	1	0	0	0	0
71147	1	0	0	0	0
71148	1	0	0	0	0
72149	1	0	0	0	0
73150	1	0	0	0	0
73151	1	0	0	0	0
73152	1	0	0	0	0
74153	1	0	0	0	0
74154	1	0	0	0	0
74155	1	0	0	0	0
75156	1	0	0	0	0
75157	1	0	0	0	0
75158	1	0	0	0	0
76159	1	0	0	0	0
77160	1	0	0	0	0
77161	1	0	0	0	0
77162	1	0	0	0	0
78163	1	0	0	0	0
78164	1	0	0	0	0
78165	1	0	0	0	0
79166	1	0	0	0	0

Belantamab mafodotin

Chemical structure

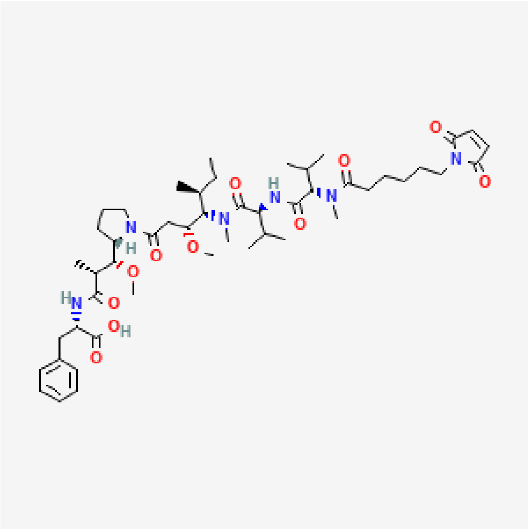

Chemical formula

C49H76N6O11

MOL file

APtclcactv10112212062D 0 0.00000 0.00000

142144 0 0 0 0 0 0 0 0999 V2000
9.9888 5.0218 0.0000 C 0 0 0 0 0 0 0 0
0 0 0 0
10.1968 4.0436 0.0000 C 0 0 0 0 0 0 0 0
0 0 0 0
9.4536 3.3745 0.0000 C 0 0 0 0 0 0 0 0
0 0 0 0
8.5026 3.6835 0.0000 C 0 0 0 0 0 0 0 0
0 0 0 0
9.6615 2.3963 0.0000 C 0 0 0 0 0 0 0 0
0 0 0 0
8.9184 1.7272 0.0000 C 0 0 0 0 0 0 0 0
0 0 0 0
7.9673 2.0362 0.0000 C 0 0 0 0 0 0 0 0
0 0 0 0
7.2242 1.3671 0.0000 C 0 0 0 0 0 0 0 0
0 0 0 0
7.4321 0.3890 0.0000 O 0 0 0 0 0 0 0 0
0 0 0 0
6.2731 1.6761 0.0000 N 0 0 0 0 0 0 0 0
0 0 0 0
5.9641 2.6272 0.0000 C 0 0 0 0 0 0 0 0
0 0 0 0
4.9641 2.6272 0.0000 C 0 0 0 0 0 0 0 0
0 0 0 0

SMILE format

CCC(C)C(C(CC(=O)N1CCCC1C(OC)C(C)
C(=O)NC(CC1=CC=CC=C1)C(O)=O)OC)N(
C)C(=O)C(NC(=O)C(C(C)C)N(C)C(=O)CC
CCCN1C(=O)C=CC1=O)C(C)C

```
4.6551 1.6761 0.0000 C 0 0 0 0 0 0 0 0 0 0 0 0
5.4641 1.0883 0.0000 C 0 0 0 0 0 0 0 0 0 0 0 0
5.4641 0.0883 0.0000 C 0 0 0 0 0 0 0 0 0 0 0 0
6.3301 -0.4117 0.0000 O 0 0 0 0 0 0 0 0 0 0 0 0
6.3301 -1.4117 0.0000 C 0 0 0 0 0 0 0 0 0 0 0 0
4.5981 -0.4117 0.0000 C 0 0 0 0 0 0 0 0 0 0 0 0
3.7321 0.0883 0.0000 C 0 0 0 0 0 0 0 0 0 0 0 0
4.5981 -1.4117 0.0000 C 0 0 0 0 0 0 0 0 0 0 0 0
5.4641 -1.9117 0.0000 O 0 0 0 0 0 0 0 0 0 0 0 0
3.7321 -1.9117 0.0000 N 0 0 0 0 0 0 0 0 0 0 0 0
3.7321 -2.9117 0.0000 C 0 0 0 0 0 0 0 0 0 0 0 0
2.8660 -3.4117 0.0000 C 0 0 0 0 0 0 0 0 0 0 0 0
2.8660 -4.4117 0.0000 C 0 0 0 0 0 0 0 0 0 0 0 0
2.0000 -4.9117 0.0000 C 0 0 0 0 0 0 0 0 0 0 0 0
2.0000 -5.9117 0.0000 C 0 0 0 0 0 0 0 0 0 0 0 0
2.8660 -6.4117 0.0000 C 0 0 0 0 0 0 0 0 0 0 0 0
3.7321 -5.9117 0.0000 C 0 0 0 0 0 0 0 0 0 0 0 0
3.7321 -4.9117 0.0000 C 0 0 0 0 0 0 0 0 0 0 0 0
4.5981 -3.4117 0.0000 C 0 0 0 0 0 0 0 0 0 0 0 0
5.4641 -2.9117 0.0000 O 0 0 0 0 0 0 0 0 0 0 0 0
4.5981 -4.4117 0.0000 O 0 0 0 0 0 0 0 0 0 0 0 0
9.1263 0.7491 0.0000 O 0 0 0 0 0 0 0 0 0 0 0 0
10.0773 0.4401 0.0000 C 0 0 0 0 0 0 0 0 0 0 0 0
10.6126 2.0873 0.0000 N 0 0 0 0 0 0 0 0 0 0 0 0
10.8205 1.1092 0.0000 C 0 0 0 0 0 0 0 0 0 0 0 0
11.3557 2.7565 0.0000 C 0 0 0 0 0 0 0 0 0 0 0 0
```

11.1478 3.7346 0.0000 O 0 0 0 0 0 0 0 0
0 0 0 0
12.3068 2.4474 0.0000 C 0 0 0 0 0 0 0 0
0 0 0 0
13.0499 3.1166 0.0000 N 0 0 0 0 0 0 0 0
0 0 0 0
14.0010 2.8076 0.0000 C 0 0 0 0 0 0 0 0
0 0 0 0
14.2089 1.8294 0.0000 O 0 0 0 0 0 0 0 0
0 0 0 0
14.7441 3.4767 0.0000 C 0 0 0 0 0 0 0 0
0 0 0 0
14.5362 4.4548 0.0000 C 0 0 0 0 0 0 0 0
0 0 0 0
15.2794 5.1240 0.0000 C 0 0 0 0 0 0 0 0
0 0 0 0
13.5852 4.7639 0.0000 C 0 0 0 0 0 0 0 0
0 0 0 0
15.6952 3.1677 0.0000 N 0 0 0 0 0 0 0 0
0 0 0 0
15.9031 2.1895 0.0000 C 0 0 0 0 0 0 0 0
0 0 0 0
16.4383 3.8368 0.0000 C 0 0 0 0 0 0 0 0
0 0 0 0
16.2304 4.8149 0.0000 O 0 0 0 0 0 0 0 0
0 0 0 0
17.3894 3.5278 0.0000 C 0 0 0 0 0 0 0 0
0 0 0 0
18.1325 4.1969 0.0000 C 0 0 0 0 0 0 0 0
0 0 0 0
19.0836 3.8879 0.0000 C 0 0 0 0 0 0 0 0
0 0 0 0
19.8267 4.5570 0.0000 C 0 0 0 0 0 0 0 0
0 0 0 0
20.7778 4.2480 0.0000 C 0 0 0 0 0 0 0 0
0 0 0 0
21.5209 4.9171 0.0000 N 0 0 0 0 0 0 0 0
0 0 0 0
22.4991 4.7092 0.0000 C 0 0 0 0 0 0 0 0
0 0 0 0
22.9058 3.7957 0.0000 O 0 0 0 0 0 0 0 0
0 0 0 0
22.9991 5.5753 0.0000 C 0 0 0 0 0 0 0 0
0 0 0 0
22.3299 6.3184 0.0000 C 0 0 0 0 0 0 0 0
0 0 0 0
21.4164 5.9117 0.0000 C 0 0 0 0 0 0 0 0
0 0 0 0
20.5504 6.4117 0.0000 O 0 0 0 0 0 0 0 0
0 0 0 0
12.5147 1.4693 0.0000 C 0 0 0 0 0 0 0 0
0 0 0 0

13.4657	1.1603	0.0000	C	0 0 0 0 0 0 0 0 0 0 0 0
11.7715	0.8002	0.0000	C	0 0 0 0 0 0 0 0 0 0 0 0
10.5953	5.1507	0.0000	H	0 0 0 0 0 0 0 0 0 0 0 0
9.8599	5.6282	0.0000	H	0 0 0 0 0 0 0 0 0 0 0 0
9.3824	4.8929	0.0000	H	0 0 0 0 0 0 0 0 0 0 0 0
10.5253	3.5178	0.0000	H	0 0 0 0 0 0 0 0 0 0 0 0
10.7716	4.2759	0.0000	H	0 0 0 0 0 0 0 0 0 0 0 0
9.3247	3.9809	0.0000	H	0 0 0 0 0 0 0 0 0 0 0 0
8.6941	4.2732	0.0000	H	0 0 0 0 0 0 0 0 0 0 0 0
7.9129	3.8751	0.0000	H	0 0 0 0 0 0 0 0 0 0 0 0
8.3110	3.0939	0.0000	H	0 0 0 0 0 0 0 0 0 0 0 0
10.1223	2.8112	0.0000	H	0 0 0 0 0 0 0 0 0 0 0 0
9.5080	1.5356	0.0000	H	0 0 0 0 0 0 0 0 0 0 0 0
8.2584	2.5837	0.0000	H	0 0 0 0 0 0 0 0 0 0 0 0
7.4788	2.4179	0.0000	H	0 0 0 0 0 0 0 0 0 0 0 0
6.5706	2.7561	0.0000	H	0 0 0 0 0 0 0 0 0 0 0 0
5.8993	3.2438	0.0000	H	0 0 0 0 0 0 0 0 0 0 0 0
5.0289	3.2438	0.0000	H	0 0 0 0 0 0 0 0 0 0 0 0
4.3577	2.7561	0.0000	H	0 0 0 0 0 0 0 0 0 0 0 0
4.0887	1.9283	0.0000	H	0 0 0 0 0 0 0 0 0 0 0 0
4.3451	1.1392	0.0000	H	0 0 0 0 0 0 0 0 0 0 0 0
6.0165	0.8069	0.0000	H	0 0 0 0 0 0 0 0 0 0 0 0
5.4641	-0.5317	0.0000	H	0 0 0 0 0 0 0 0 0 0 0 0
5.7101	-1.4117	0.0000	H	0 0 0 0 0 0 0 0 0 0 0 0
6.3301	-2.0317	0.0000	H	0 0 0 0 0 0 0 0 0 0 0 0
6.9501	-1.4117	0.0000	H	0 0 0 0 0 0 0 0 0 0 0 0

4.5981 0.2083 0.0000 H 0 0 0 0 0 0 0 0
0 0 0 0
4.0421 0.6253 0.0000 H 0 0 0 0 0 0 0 0
0 0 0 0
3.1951 0.3983 0.0000 H 0 0 0 0 0 0 0 0
0 0 0 0
3.4221 -0.4486 0.0000 H 0 0 0 0 0 0 0 0
0 0 0 0
3.1951 -1.6017 0.0000 H 0 0 0 0 0 0 0 0
0 0 0 0
4.2690 -2.6017 0.0000 H 0 0 0 0 0 0 0 0
0 0 0 0
2.6540 -2.8291 0.0000 H 0 0 0 0 0 0 0 0
0 0 0 0
2.2554 -3.5193 0.0000 H 0 0 0 0 0 0 0 0
0 0 0 0
1.4631 -4.6017 0.0000 H 0 0 0 0 0 0 0 0
0 0 0 0
1.4631 -6.2217 0.0000 H 0 0 0 0 0 0 0 0
0 0 0 0
2.8660 -7.0317 0.0000 H 0 0 0 0 0 0 0 0
0 0 0 0
4.2690 -6.2217 0.0000 H 0 0 0 0 0 0 0 0
0 0 0 0
4.2690 -4.6017 0.0000 H 0 0 0 0 0 0 0 0
0 0 0 0
6.0010 -3.2217 0.0000 H 0 0 0 0 0 0 0 0
0 0 0 0
9.8858 -0.1496 0.0000 H 0 0 0 0 0 0 0 0
0 0 0 0
10.6670 0.2485 0.0000 H 0 0 0 0 0 0 0 0
0 0 0 0
10.2689 1.0297 0.0000 H 0 0 0 0 0 0 0 0
0 0 0 0
10.2140 0.9803 0.0000 H 0 0 0 0 0 0 0 0
0 0 0 0
10.9494 0.5027 0.0000 H 0 0 0 0 0 0 0 0
0 0 0 0
11.4269 1.2381 0.0000 H 0 0 0 0 0 0 0 0
0 0 0 0
12.1779 3.0539 0.0000 H 0 0 0 0 0 0 0 0
0 0 0 0
12.9210 3.7230 0.0000 H 0 0 0 0 0 0 0 0
0 0 0 0
14.8730 2.8702 0.0000 H 0 0 0 0 0 0 0 0
0 0 0 0
15.1259 4.2632 0.0000 H 0 0 0 0 0 0 0 0
0 0 0 0
15.6942 4.6632 0.0000 H 0 0 0 0 0 0 0 0
0 0 0 0
15.7401 5.5388 0.0000 H 0 0 0 0 0 0 0 0
0 0 0 0

14.8645 5.5847 0.0000 H 0 0 0 0 0 0 0 0
0 0 0 0
13.7767 5.3535 0.0000 H 0 0 0 0 0 0 0 0
0 0 0 0
12.9955 4.9554 0.0000 H 0 0 0 0 0 0 0 0
0 0 0 0
13.3936 4.1742 0.0000 H 0 0 0 0 0 0 0 0
0 0 0 0
15.2966 2.0606 0.0000 H 0 0 0 0 0 0 0 0
0 0 0 0
16.0320 1.5831 0.0000 H 0 0 0 0 0 0 0 0
0 0 0 0
16.5095 2.3184 0.0000 H 0 0 0 0 0 0 0 0
0 0 0 0
17.0983 2.9804 0.0000 H 0 0 0 0 0 0 0 0
0 0 0 0
17.8780 3.1461 0.0000 H 0 0 0 0 0 0 0 0
0 0 0 0
18.4236 4.7443 0.0000 H 0 0 0 0 0 0 0 0
0 0 0 0
17.6440 4.5786 0.0000 H 0 0 0 0 0 0 0 0
0 0 0 0
18.7925 3.3405 0.0000 H 0 0 0 0 0 0 0 0
0 0 0 0
19.5722 3.5062 0.0000 H 0 0 0 0 0 0 0 0
0 0 0 0
20.1178 5.1045 0.0000 H 0 0 0 0 0 0 0 0
0 0 0 0
19.3382 4.9387 0.0000 H 0 0 0 0 0 0 0 0
0 0 0 0
20.4867 3.7006 0.0000 H 0 0 0 0 0 0 0 0
0 0 0 0
21.2664 3.8663 0.0000 H 0 0 0 0 0 0 0 0
0 0 0 0
23.6157 5.6401 0.0000 H 0 0 0 0 0 0 0 0
0 0 0 0
22.4589 6.9249 0.0000 H 0 0 0 0 0 0 0 0
0 0 0 0
11.9250 1.6609 0.0000 H 0 0 0 0 0 0 0 0
0 0 0 0
13.2742 0.5706 0.0000 H 0 0 0 0 0 0 0 0
0 0 0 0
14.0554 0.9687 0.0000 H 0 0 0 0 0 0 0 0
0 0 0 0
13.6573 1.7499 0.0000 H 0 0 0 0 0 0 0 0
0 0 0 0
11.3567 1.2609 0.0000 H 0 0 0 0 0 0 0 0
0 0 0 0
11.3108 0.3853 0.0000 H 0 0 0 0 0 0 0 0
0 0 0 0
12.1864 0.3394 0.0000 H 0 0 0 0 0 0 0 0
0 0 0 0

1	2	1	0	0	0	0
2	3	1	0	0	0	0
3	4	1	0	0	0	0
3	5	1	0	0	0	0
5	6	1	0	0	0	0
6	7	1	0	0	0	0
7	8	1	0	0	0	0
8	9	2	0	0	0	0
8	10	1	0	0	0	0
10	11	1	0	0	0	0
11	12	1	0	0	0	0
12	13	1	0	0	0	0
13	14	1	0	0	0	0
10	14	1	0	0	0	0
14	15	1	0	0	0	0
15	16	1	0	0	0	0
16	17	1	0	0	0	0
15	18	1	0	0	0	0
18	19	1	0	0	0	0
18	20	1	0	0	0	0
20	21	2	0	0	0	0
20	22	1	0	0	0	0
22	23	1	0	0	0	0
23	24	1	0	0	0	0
24	25	1	0	0	0	0
25	26	2	0	0	0	0
26	27	1	0	0	0	0
27	28	2	0	0	0	0
28	29	1	0	0	0	0
29	30	2	0	0	0	0
25	30	1	0	0	0	0
23	31	1	0	0	0	0
31	32	1	0	0	0	0
31	33	2	0	0	0	0
6	34	1	0	0	0	0
34	35	1	0	0	0	0
5	36	1	0	0	0	0
36	37	1	0	0	0	0
36	38	1	0	0	0	0
38	39	2	0	0	0	0
38	40	1	0	0	0	0
40	41	1	0	0	0	0
41	42	1	0	0	0	0
42	43	2	0	0	0	0
42	44	1	0	0	0	0
44	45	1	0	0	0	0
45	46	1	0	0	0	0
45	47	1	0	0	0	0
44	48	1	0	0	0	0
48	49	1	0	0	0	0
48	50	1	0	0	0	0
50	51	2	0	0	0	0

50 52 1 0 0 0 0
52 53 1 0 0 0 0
53 54 1 0 0 0 0
54 55 1 0 0 0 0
55 56 1 0 0 0 0
56 57 1 0 0 0 0
57 58 1 0 0 0 0
58 59 2 0 0 0 0
58 60 1 0 0 0 0
60 61 2 0 0 0 0
61 62 1 0 0 0 0
57 62 1 0 0 0 0
62 63 2 0 0 0 0
40 64 1 0 0 0 0
64 65 1 0 0 0 0
64 66 1 0 0 0 0
1 67 1 0 0 0 0
1 68 1 0 0 0 0
1 69 1 0 0 0 0
2 70 1 0 0 0 0
2 71 1 0 0 0 0
3 72 1 0 0 0 0
4 73 1 0 0 0 0
4 74 1 0 0 0 0
4 75 1 0 0 0 0
5 76 1 0 0 0 0
6 77 1 0 0 0 0
7 78 1 0 0 0 0
7 79 1 0 0 0 0
11 80 1 0 0 0 0
11 81 1 0 0 0 0
12 82 1 0 0 0 0
12 83 1 0 0 0 0
13 84 1 0 0 0 0
13 85 1 0 0 0 0
14 86 1 0 0 0 0
15 87 1 0 0 0 0
17 88 1 0 0 0 0
17 89 1 0 0 0 0
17 90 1 0 0 0 0
18 91 1 0 0 0 0
19 92 1 0 0 0 0
19 93 1 0 0 0 0
19 94 1 0 0 0 0
22 95 1 0 0 0 0
23 96 1 0 0 0 0
24 97 1 0 0 0 0
24 98 1 0 0 0 0
26 99 1 0 0 0 0
27100 1 0 0 0 0
28101 1 0 0 0 0
29102 1 0 0 0 0

```
30103  1  0  0  0  0
32104  1  0  0  0  0
35105  1  0  0  0  0
35106  1  0  0  0  0
35107  1  0  0  0  0
37108  1  0  0  0  0
37109  1  0  0  0  0
37110  1  0  0  0  0
40111  1  0  0  0  0
41112  1  0  0  0  0
44113  1  0  0  0  0
45114  1  0  0  0  0
46115  1  0  0  0  0
46116  1  0  0  0  0
46117  1  0  0  0  0
47118  1  0  0  0  0
47119  1  0  0  0  0
47120  1  0  0  0  0
49121  1  0  0  0  0
49122  1  0  0  0  0
49123  1  0  0  0  0
52124  1  0  0  0  0
52125  1  0  0  0  0
53126  1  0  0  0  0
53127  1  0  0  0  0
54128  1  0  0  0  0
54129  1  0  0  0  0
55130  1  0  0  0  0
55131  1  0  0  0  0
56132  1  0  0  0  0
56133  1  0  0  0  0
60134  1  0  0  0  0
61135  1  0  0  0  0
64136  1  0  0  0  0
65137  1  0  0  0  0
65138  1  0  0  0  0
65139  1  0  0  0  0
66140  1  0  0  0  0
66141  1  0  0  0  0
66142  1  0  0  0  0
M  END
$$$$
```
